# Occurrence of *Salmonella* spp. in animal patients and the hospital environment at a veterinary academic hospital in South Africa

**DOI:** 10.14202/vetworld.2024.922-932

**Published:** 2024-04-29

**Authors:** Ayesha Bibi Karodia, Tahiyya Shaik, Daniel Nenene Qekwana

**Affiliations:** Department of Paraclinical Sciences, Section Veterinary Public Health, University of Pretoria, Pretoria, Gauteng, South Africa

**Keywords:** environment, hospital, animals, risk factors, *Salmonella enterica*, Typhimurium, veterinary

## Abstract

**Background and Aims:**

Nosocomial infections caused by *Salmonella* spp. are common in veterinary facilities. The early identification of high-risk patients and sources of infection is important for mitigating the spread of infections to animal patients and humans. This study investigated the occurrence of *Salmonella* spp. among patients at a veterinary academic hospital in South Africa. In addition, this study describes the environmental factors that contribute to the spread of *Salmonella* spp. in the veterinary facility.

**Materials and Methods:**

This study used a dataset of *Salmonella-*positive animals and environmental samples submitted to the bacteriology laboratory between 2012 and 2019. The occurrence of *Salmonella* isolates at the veterinary hospital was described based on source, month, season, year, and location. Proportions and 95% confidence intervals were calculated for each variable.

**Results:**

A total of 715 *Salmonella* isolates were recorded, of which 67.6% (483/715) came from animals and the remainder (32.4%, 232/715) came from environmental samples. The highest proportion (29.2%) of *Salmonella* isolates was recorded in 2016 and most isolates were reported in November (17.4%). The winter season had the lowest (14.6%) proportion of isolates reported compared to spring (31.3%), summer (27.8%), and autumn (26.4%). *Salmonella* Typhimurium (20.0%) was the most frequently reported serotype among the samples tested, followed by *Salmonella* Anatum (11.2%). Among the positive animal cases, most (86.3%) came from equine clinics. Most reported isolates differed based on animal species with *S*. Typhimurium being common in equines and *S*. Anatum in bovines.

**Conclusion:**

In this study, *S*. Typhimurium emerged as the predominant strain in animal and environmental samples. Equines were the most affected animals; however, *Salmonella* serotypes were also detected in the production animals. Environmental contamination was also a major source of *Salmonella* species in this study. To reduce the risk of transmission, strict infection prevention and control measures (biosecurity) must be implemented.

## Introduction

Salmonellosis is an infectious bacterial disease caused by *Salmonella* Enterica or *Salmonella* Bongori species [1–7]. Most serovars associated with diseases in livestock, companion animals, and wildlife belong to the *S*. Enterica subspp. [1, 8–10]. These *Salmonella* serovars have a host preference [11–14]. An example of this preference is *Salmonella* Enteritidis in poultry, *Salmonella* Typhimurium in horses [15–18], and *Salmonella* Anatum mainly in beef and *Salmonella* Weltevreden in seafood [16, 19–22].

Transmission can occur horizontally or vertically in animals [12–27]. Horizontal transmission occurs primarily through the fecal-oral route, which is facilitated by infected or contaminated animals or humans as well as fomites, water sources, and feed [12, 28]. Horses, cattle, sheep, poultry, and domestic animals are susceptible to this mode of infection and remain the predominant mode of transmission among animals [12, 29]. On the other hand, vertical transmission has been reported in poultry [25, 26] and cattle [23, 30]. The risk of *Salmonella* infection in animals is strongly associated with increased stress, exposure to antimicrobials, age, sex [31], season [19, 28], and host susceptibility [28].

Many *Salmonella*-infected animals are asymptomatic and intermittent shedders. However, clinical signs such as pyrexia, diarrhea, anorexia, and colic in equines, vomiting in cats and dogs [32, 33], and abortion in certain species such as sheep have been reported [34]. High morbidity and mortality [35], particularly with multidrug-resistant *Salmonella* spp. [9, 36], are major concerns.

Diagnosis of salmonellosis is based on the clinical signs and laboratory confirmation [37], including culture [38, 39], polymerase chain reaction (PCR) [40–42], serum agglutination testing, and enzyme-linked immunosorbent assays [43]. However, serological tests are less sensitive compared to PCR [43].

Treatment of infected animals depends on the severity of the disease and can be expensive and unrewarding [44]. Antimicrobial therapy is not initially recommended, but anti-inflammatory agents are preferred in most cases [28, 45–47]. In high-risk patients such as calves and foals, aggressive treatment may be required [45]. Probiotics and prebiotics have been shown to be beneficial in the prevention and treatment of salmonellosis in poultry [48–51], but their efficacy in other animals is not well documented.

Sporadic outbreaks associated with *Salmonella* spp. in both humans and animals have been reported [44, 52–55]. This is due to the persistence of *Salmonella* in the environment and continuous shedding by asymptomatic animals [35, 56, 57]. In addition, fomites, contaminated water, and feed have been identified as sources of infection [12, 58]. A contaminated environment remains the main contributor to outbreaks related to *Salmonella* spp. in veterinary facilities [10, 59]. Therefore, identifying asymptomatic animals remains crucial to prevent the transmission of infection to other animals [11, 60, 61] and their owners [11].

Research on salmonellosis in the field of veterinary medicine in developing countries is limited, particularly in South Africa. This study examined the occurrence and characteristics of *Salmonella* spp. identified in animal patients and hospital environments at a veterinary academic hospital (VAH). This study aimed to shed light on the temporal distribution of *Salmonella* spp. and the most affected animal species.

## Materials and Methods

### Ethical approval

The Faculty of Veterinary Science Research Ethics Committee and the Faculty of Humanities Research Ethics Committee (Project number: REC151-20) approved this study.

### Study period and location

The data were analyzed from June 2022 to May 2023. This study used a secondary dataset of *Salmonella*-positive cases presented at the VAH in Pretoria, South Africa. The academic hospital provides training, clinical, and diagnostic services. The hospital is divided into three sections, namely, the equine clinic, the small animal clinic (for domestic canines and felines), and the production animal clinic, in which farm animals such as bovine, ovine, and porcine are treated. It provides both routine general care and specialized services in the fields of surgery, medicine, and reproduction for different species.

### Data source

The dataset used in this study comprised both animal and environmental isolates from samples submitted to the Agricultural Research Council-Onderstepoort Veterinary Research (ARC-OVR) bacteriology laboratory of the Veterinary Hospital for routine surveillance and diagnosis between 2012 and 2019. For each positive *Salmonella* result, the following information was extracted: isolated *Salmonella* serotype, animal species, collection date, and hospital location.

*Salmonella* spp. were cultured following the procedures outlined by Gelaw *et al*. [15] and Kidanemariam *et al*. [62]: Briefly, fecal (animal samples) and environmental samples were added to buffered peptone water (pH 7.2) and incubated at 37°C for 18–24 h. One milliliter of this solution was then transferred into 9 mL of Rappaport Vassiliadis (Oxoid®, Hampshire, England) enrichment broth and incubated at 42°C for 18–24 h. subcultures from enrichment media were cultivated on selective solid media such as xylose-lysine deoxycholate agar (Difco®, e Point du Claix, France) and incubated at 37°C for 18–24 h. Black colonies with a pink periphery were preliminarily identified as *Salmonella* and further confirmed by various biochemical tests. Gram-negative isolates meeting specific criteria such as indole-negative, motile, Simmon’s citrate-negative, urease-negative, hydrogen sulfide-producing in a triple sugar iron slant, lysine decarboxylase-positive, dulcitol-fermenting but lactose-nonfermenting, and malonate-negative were classified as *S*. Enterica. Additional carbohydrate fermentation tests, including gas production in Durham tubes and fermentation of sorbitol, arabinose, rhamnose, maltose, and trehalose, were performed to identify *Salmonella* organisms that did not meet the above criteria. The *Salmonella* spp. were serotyped according to the Kauffmann–White classification scheme using a battery of polyvalent and monovalent somatic O and flagellar H antisera.

Environmental samples included stables, offices, corridors, theaters, examination areas, and storage areas. Sampling was performed using new dry-cleaning cloths by wiping 80% of the surface area before placing them in labeled sterile bags. Operators wore gloves during sampling, and cloths were attached to sterilized mops between samples. Before 2016, sampling was conducted annually, and the last annual swab was performed in February 2016. The outbreak occurred at the end of 2016 and sampling was subsequently shifted to a biannual frequency, and five random swabs were collected every month.

### Biosecurity

In general, the equine hospital performs routine fecal sampling of patients on admission and on Mondays, Thursdays and after discharge as part of routine surveillance for biosecurity reasons. Equine stables can only be reused after disinfection as well as after a negative culture of the previous patient. If a previous patient has tested positive, the stables will be disinfected and tested until they are negative. Daily cleaning with deep cleaning of surfaces is performed weekly. In addition, there is a foot bath at all entrances and exits of the clinic.

### Data management

The data were stored in Microsoft Excel (Microsoft 365, Microsoft Office, Washington, USA). Before analyses, the dataset was evaluated for missing information as well as implausible values. Final variables included year, month, season, animal species, hospital location, and Salmonella serotype that were used in the final analysis. All variables 1% were categorized as “all others.” The seasons were divided into summer (November–March), autumn (April–May), winter (June–August), and spring (September–October).

### Statistical analysis

The statistical analysis was performed using JASP version 0.16.1.0 (https://jasp-stats.org/previous-versions/). Descriptive analyses were performed to determine the proportions of *Salmonella* isolates based on serotype, animal species, month, season, and year as well as the location of the animal in the veterinary hospital. The proportions of *Salmonella* serotypes from environmental samples were also analyzed by month, season, year, and location in the veterinary hospital. If necessary, 95% confidence intervals were calculated for the variables.

## Results

### *Salmonella spp*. from environmental and animal samples

A total of 715 *Salmonella* cases were identified, of which 67.6% originated from animal sources and 32.4% from environmental sources. The highest proportion of isolates (29.2%) was reported in 2016, followed by 2014 (18.9%). Most isolates were reported in November (17.4%) and February (14.6%), with the highest peak occurring in November 2016 (Figure-1 and Table-1). However, 31.2% of *Salmonella* cases were recorded in spring, while only 14.5% were isolated in winter (Table-1).

**Figur-1:**
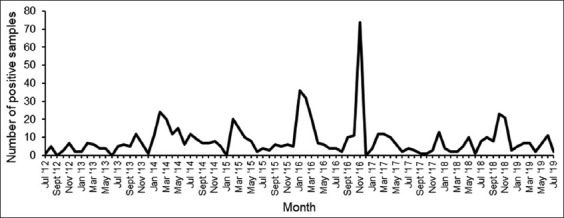
Monthly distribution of *Salmonella spp*. at a veterinary academic hospital between July 2012 and August 2019.

**Table-1:** Distribution of *Salmonella* spp. based on year, month, season, and source at a veterinary academic hospital between 2012 and 2019.

Factor	Frequency	Proportion (%)	CI
Source (n = 715)			
Animals	483	67.6	64–70
Environmental	232	32.4	29–35
Year (n = 713)			
2012	18	2.5	2–4
2013	59	8.3	6–11
2014	135	18.9	16–23
2015	84	11.8	10–14
2016	208	29.2	26–33
2017	71	10.0	8–12
2018	97	13.6	11–16
2019	41	5.8	4–8
Month (n = 713)			
January	62	8.7	7–11
February	104	14.6	12–17
March	83	11.6	9–14
April	50	7.0	5–9
May	56	7.9	6–10
June	26	3.6	3–5
July	40	5.6	4–8
August	37	5.2	4–7
September	37	5.2	4–7
October	62	8.7	7–11
November	124	17.4	15–20
December	32	4.5	3–6
Season (n = 713)			
Autumn	188	26.4	23–30
Winter	104	14.6	12–17
Summer	198	27.8	25–31
Spring	223	31.3	28–35

CI: Confidence interval

The most common serotypes among all samples were *S*. Typhimurium (20%), followed by *S*. Anatum (11.2%) and *Salmonella* Polyvalent OMD (5.7%) (Table-2).

**Table-2:** *Salmonella* spp. from animal and environmental samples recorded by the bacteriology laboratory between 2012 and 2019 (n = 715).

Serotypes	Frequency	Proportion (%)	CI
*S.* Typhimurium	143	20	17–23
*S.* Anatum	80	11.2	9–14
*Salmonella* Polyvalent OMD	41	5.7	4–8
*Salmonella* Polyvalent OD	35	4.9	4–7
*S.* Heidelberg	32	4.5	3–6
*S.* Infantis	31	4.3	3–6
*Salmonella* Polyvalent OMC	28	3.9	3–6
*S.* Bovismorbificans	16	2.2	1–4
*S.* Muenchen	15	2.1	1–3
*S.* Enteritidis	14	2	1–3
*S.* Braenderup	9	1.3	1–2
*S.* Meleagridis	9	1.3	1–2
*S.* Irumu	8	1.1	1–2
*S.* Pretoria	8	1.1	1–2
*S.* Virchow	8	1.1	1–2
*Salmonella* II	8	1.1	1–2
*Salmonella* Polyvalent OE	8	1.1	1–2
Untyped	8	1.1	1–2
All others	214	29.9	27–33

CI: Confidence interval, S. Typhimurium=*Salmonella* Typhimurium, S. Anatum=*Salmonella* Anatum, S. Heidelberg=*Salmonella* Heidelberg, S. Infantis=*Salmonella* Infantis, S. Bovismorbificans=*Salmonella* Bovismorbificans, S. Muenchen=*Salmonella* Muenchen, S. Enteritidis=*Salmonella* Enteritidis, S. Braenderup=*Salmonella* Braenderup, S. Meleagridis=*Salmonella* Meleagridis, S. Irumu=*Salmonella* Irumu, S. Pretoria=*Salmonella* Pretoria, S. Virchow=*Salmonella* Virchow, *Salmonella* Polyvalent OMD=Antiserum O mixture of group D, *Salmonella* Polyvalent OD=Antiserum O group D, *Salmonella* Polyvalent OMC=Antiserum O mixture of group C

#### Salmonella serotypes isolated from animal samples

Among the animal isolates (n = 483), 86.3% and 13.6% were from the equine and production animal clinics, respectively. No *Salmonella* cases have been reported in animal samples collected from small animal clinics. The majority (86.1%) of *Salmonella* organisms came from equines, followed by bovines (7%) and ovines (3.3%) (Table-3).

**Table-3:** Distribution of *Salmonella* isolates recorded by the bacteriology laboratory based on the clinic of origin and animal species affected, 2012–2019.

Source	Frequency	Percentage	CI^a^
Animal clinic			
Equine	417	86.3	83–89
Production	66	13.7	11–17
Animal species			
Bovine	34	7.0	5–10
Camel	1	0.2	0–1
Caprine	8	1.7	1–3
Equine	416	86.1	83–89
Ovine	16	3.3	2–5
Porcine	4	0.8	0–2
Rhino	4	0.8	0–2

a CI=Confidence interval

Equines and bovines

In equines, reported serotypes included *S*. Typhimurium (18.8%), *S*. Anatum (10.1%), *Salmonella* Polyvalent OD (5.3%), and *S*. Infantis (5%). The most common serotype among bovine *Salmonella* isolates (n = 34) was *S*. Anatum (23.5%) followed by *S*. Typhimurium (14.7%) (Table-4).

**Table-4:** Distribution of *Salmonella* spp. among equine and bovine samples from the veterinary academic hospital between 2012 and 2019.

Animal species	Serotypes	Frequency	Proportion (%)
Equines	*S.* Typhimurium	78	18.8
	*S.* Anatum	42	10.1
	*Salmonella* Polyvalent OD	22	5.3
	*S.* Infantis	21	5.0
	*Salmonella* Polyvalent OMC	17	4.1
	*Salmonella* Polyvalent OMD	15	3.6
	*S.* Bovismorbificans	13	3.1
	*S.* Heidelberg	12	2.9
	*S.* Enteritidis	11	2.6
	*S.* Muenchen	10	2.4
	Untypeable/Untypeable	9	2.2
	*S.* Braenderup	7	1.7
	*S.* Pretoria	7	1.7
	*S.* Virchow	7	1.7
	*S.* Kottbus	6	1.4
	*S.* Abaetetuba	5	1.2
	*S.* Kibusi	5	1.2
	All others	129	31.0
Bovines	*S.* Anatum	8	23.5
	*S.* Typhimurium	5	14.7
	*S.* Infantis	3	8.8
	*S.* Muenchen	3	8.8
	*Salmonella* II	3	8.8
	*Salmonella* Polyvalent OD	2	5.9
	*S.* Bovismorbificans	1	2.9
	*S.* Braenderup	1	2.9
	*S.* Dublin	1	2.9
	*S.* Fulda	1	2.9
	*S.* Hadar	1	2.9
	*S.* Mikawasima	1	2.9
	*S.* Nottingham	1	2.9
	*S.* Tennessee	1	2.9
	*S.* Wangata	1	2.9

S. Typhimurium=*Salmonella* Typhimurium, S. Anatum=*Salmonella* Anatum, S. Infantis=*Salmonella* Infantis, S. Bovismorbificans=*Salmonella* Bovismorbificans, S. Heidelberg=*Salmonella* Heidelberg, S. Enteritidis=*Salmonella* Enteritidis, S. Muenchen=*Salmonella* Muenchen, S. Braenderup=*Salmonella* Braenderup, Pretoria=*Salmonella* Pretoria, S. Virchow=*Salmonella* Virchow, S. Kottbus=*Salmonella* Kottbus, S. Abaetetuba=*Salmonella* Abaetetuba, S. Kibusi=*Salmonella* Kibusi, S. Dublin=*Salmonella* Dublin, S. Fulda=*Salmonella* Fulda*,* S. Hadar=*Salmonella* Hadar, S. Mikawasima=*Salmonella* Mikawasima, S. Nottingham=S*almonella* Nottingham, S. Tennessee=*Salmonella* Tennessee, S. Wangata=*Salmonella* Wangata

Camelids, caprines, ovines, porcines, and rhinoceros

Several *Salmonella* serovars were also identified from camelids, caprines, ovines, porcines, and rhinoceros samples (Table-5).

**Table-5:** Distribution of *Salmonella* spp. in camelids caprines, ovines, porcines, and rhinoceros samples from the veterinary academic hospital between 2012 and 2019.

Animal species	Serotypes	Frequency	Percentage
Camelid	*S.* Infantis	1	100.0
Caprine	*Salmonella* Polyvalent OMD	2	25.0
	*S.* Anatum	1	12.5
	*S.* Infantis	1	12,5
	*S.* Livingstone	1	12.5
	*S.* Minnesota	1	12.5
	*S.* Newport	1	12.5
	*Salmonella* Polyvalent OMC	1	12.5
Ovine	*Salmonella* Polyvalent OE	3	18.8
	*Salmonella* Polyvalent OMC	2	12.5
	*Salmonella* Polyvalent OMD	2	12.5
	*S.* Agona	1	6.3
	*S.* Anatum	1	6.3
	*S.* Braenderup	1	6.3
	*S.* Fillmore	1	6.3
	*S.* Schwarzengrund	1	6.3
	*Salmonella* Group D	1	6.3
	*Salmonella* Polyvalent OD	1	6.3
	*Salmonella* Polyvalent OME	1	6.3
	Untypeable	1	6.3
Porcine	*S.* Enteritidis	1	25.0
	*S.* Fulda	1	25.0
	*S.* Heidelberg	1	25.0
	*S.* Sculocoates	1	25.0
Rhinoceros	*S.* Othmarschen	1	25.0
	*S.* Typhimurium	1	25.0
	*Salmonella* Polyvalent OD	1	25.0
	*Salmonella* Polyvalent OE	1	25.0

S. Infantis=*Salmonella* Infantis, S. Anatum=*Salmonella* Anatum, S. Livingstone=*Salmonella* Livingstone, S. Minnesota=*Salmonella* Minnesota,* S.* Newport=*Salmonella* Newport, S. Agona=*Salmonella* Agona, S. Braenderup=*Salmonella* Braenderup, S. Fillmore=*Salmonella* Fillmore, S. Schwarzengrund=*Salmonella* Schwarzengrund, S. Enteritidis=*Salmonella* Enteritidis, S. Fulda=*Salmonella* Fulda, S. Heidelberg=*Salmonella* Heidelberg, S. Sculocoates=*Salmonella* Sculocoates, S. Othmarschen=*Salmonella* Othmarschen, S. Typhimurium=*Salmonella* Typhimurium

**Figure-2:**
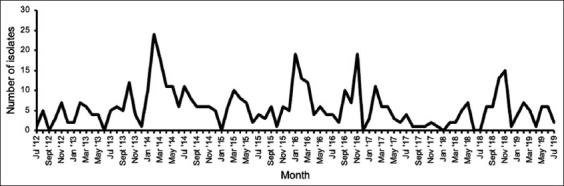
Monthly distribution of Salmonella-positive animals at the veterinary academic hospital between 2012 and 2019.

Four peaks in the number of *Salmonella*-positive animals were observed in February 2014, January 2016, November 2016, and November 2018 (Figure-2).

#### Salmonella serotypes isolated from environmental samples

Among the environmental isolates, the majority were from the equine clinic (62.5%, 145/232), followed by the production animal clinic (37.1%, 86/232), and the small animal hospital (0.4%, 1/232). The most frequently reported serotypes were *S*. Typhimurium (25.4%), *S*. Anatum (12.1%), *Salmonella* polyvalent OMD (9.5%), and *S*. Heidelberg (8.2%; Table-6).

**Table-6:** Description of *Salmonella* spp. from environmental samples tested at the veterinary academic hospital between 2012 and 2019.

Serotype	Frequency	Percentage
*S.* Typhimurium	59	25.4
*S*. Anatum	28	12.1
*Salmonella* Polyvalent OMD	22	9.5
*S.* Heidelberg	19	8.2
*Salmonella* Polyvalent OD	9	3.9
*S*. Meleagridis	8	3.4
*Salmonella* Polyvalent OMC	8	3.4
*S.* Infantis	5	2.2
*S.* Irumu	4	1.7
*S*. Newport	4	1.7
*S*. Bovismorbificans	3	1.3
*S.* Colorado	3	1.3
*S*. Fulda	3	1.3
*S*. Korlebu	3	1.3
*S.* Schwarzengrund	3	1.3
*Salmonella* Group C1	3	1.3
Total	232	100

S. Typhimurium=*Salmonella* Typhimurium, S. Anatum*=Salmonella* Anatum, S. Heidelberg*=Salmonella* Heidelberg, S. Meleagridis=*Salmonella* Meleagridis, S. Infantis=*Salmonella* Infantis, S. Irumu=*Salmonella* Irumu, S. Newport=*Salmonella* Newport, S. Bovismorbificans=*Salmonella* Bovismorbificans, S. Colorado=*Salmonella* Colorado, S. Fulda=*Salmonella* Fulda, S. Korlebu=*Salmonella* Korlebu, S. Schwarzengrund=*Salmonella* Schwarzengrund

**Figure-3:**
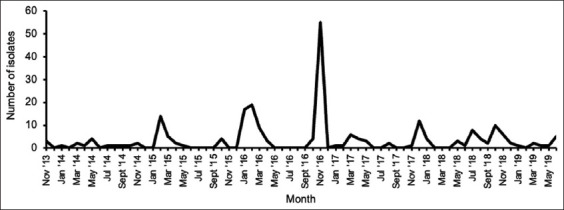
Distribution of environmental *Salmonella* isolates received by month from July 2012 to August 2019 at Onderstepoort Veterinary Academic Hospital in Pretoria, South Africa.

Among the environmental samples, the highest peak in *Salmonella* cases was reported in November 2016 (Figure-3).

## Discussion

*Salmonella* cases in veterinary hospitals are often nosocomial [63–66] and are usually associated with environmental contamination [67]. This study focuses on *Salmonella* spp. isolated from veterinary patients and hospital environments. The majority of *Salmonella* serotypes were isolated from environmental samples collected from the equine section of the hospital. This is unsurprising, since equines have been described as intermittent shedders of *Salmonella* spp., which play a significant role in environmental contamination [15, 63, 67, 68]. On the other hand, a study conducted at Ohio State University reported a higher number of environmental *Salmonella* cases from livestock compared to equines [69]. The highest number of *Salmonella* cases has been reported in warmer seasons, suggesting favorable climatic conditions supporting bacterial spread [19–74]. Similar to other studies [62, 68, 75–78], this study observed seasonal patterns in the number of *Salmonella* cases, emphasizing the need for increased biosecurity and infection control measures to minimize the spread of bacteria in the hospital setting during warmer period.

### *Salmonella* serotypes isolated from animals

*S*. Typhimurium was the most reported serotype among animals in this study, similar to the findings of two South African studies [62, 79]. However, another South African study by Gelaw *et al*. [15] reported that *S*. Heidelberg is the most common serotype isolated from animals, demonstrating potential variations in serotype distribution based on study populations. Among the equids, *S*. Typhimurium was the most common type, followed by *S*. Anatum and *S*. Heidelberg. Other studies have also reported that *S*. Typhimurium is the most frequent serotype in equines [58, 63, 67, 80]. On the contrary, in 2008, the most common serotype among the equines in South Africa was *S*. Heidelberg, followed by *S*. Anatum and *S*. Typhimurium [15]. In bovines, *S*. Anatum was the most common serotype, which is similar to the findings of the previous study [16]. On the other hand, two South African studies [15, 62] reported that *Salmonella* Dublin followed by *S*. Anatum is the most common serotype. Globally, *S*. Typhimurium and *Salmonella* Montevideo were the most reported serotype in bovines [81–86]. Other *Salmonella* serotypes were reported in ovine, caprine, porcine, rhinoceros, and camelid animals in this study, albeit less often. *Salmonella* spp. have also been reported in porcines [60, 87–90], ovines [48, 91–94], caprines [95–99], camelids [100–106], and a lesser extent in rhinoceros [107–111]. Notably, no *Salmonella* spp. were observed in cats and dogs during the study period, which is consistent with the findings of other studies [112–114]. However, a study conducted at the same hospital in 2017 suggested that *Salmonella* spp. circulate among apparently healthy and sick companion animals [115]. Regular screening in both apparently healthy and clinical cases may provide valuable insights into the distribution of *Salmonella* spp. among companion animals presented at VAHs.

### *Salmonella* serotypes from the environment

*Salmonella* serotypes isolated from environmental samples in this study mirrored the patterns observed in animal samples [63, 67]. For example, *S*. Typhimurium is commonly isolated in equines, while *S*. Anatum is common in bovines. Some studies have reported differences in the profile of *Salmonella* spp. isolated from environmental samples compared to animal samples [69, 114, 116]. The high correlation between environmental and animal samples can be attributed to environmental contamination by both asymptomatic and symptomatic shedders [72, 117, 118]. In addition, the presence of *S*. Typhimurium in high proportions compared to other organisms could be due to its increased resistance to disinfection, allowing it to persist longer in the environment [119–121].

Several serotypes not reported in animals, including *Salmonella* Adeyo, *Salmonella* Aschersleben, *Salmonella* Berta, and *Salmonella* Blegdom, were identified in the environmental samples in this study. These findings suggest that other potential sources in the hospital environment, such as fomites, visitors, wildlife, rodents, birds, and insects [9, 114, 122], might play a role. However, further studies are needed to understand the clinical significance.

Although biosecurity measures are rigorously implemented at veterinary hospitals, their effectiveness must be regularly evaluated [123, 124]. In addition, hospitals must consider implementing educational initiatives for veterinary staff, pet owners, and visitors to enhance awareness on the risk of *Salmonella* transmission and potential preventive measures that can be implemented [123, 125].

## Limitations

The authors did not have control over the collection process due to historical data being used in this study. Furthermore, this study focused on *Salmonella* isolates from a single laboratory at a single veterinary hospital; therefore, the results may not be representative of all veterinary facilities in South Africa. Nonetheless, the findings from this study contribute to a better understanding of the epidemiology of salmonellosis in veterinary facilities in South Africa.

## Conclusion

*Salmonella* spp. are common among animal and environmental sources in VAHs. Although *S*. Typhimurium was the most frequently reported serotype among patients and environmental samples in this study, other serotypes of zoonotic and clinical relevance were also reported. Compared with other areas, the environment in the equine area of the hospital seems to be an important source of *Salmonella*. More routine animal and environmental screening needs to be considered around this area of the hospital. Furthermore, the potential role of human carriers, including staff, students, and visitors, in the transmission of *Salmonella* should be investigated. Biosecurity measures aimed at mitigating the risk of *Salmonella* transmission in veterinary facilities should be maintained throughout the year, with further measures being implemented in warmer months. To effectively manage and prevent the transmission of *Salmonella* in veterinary hospitals, a multifaceted approach involving enhanced biosecurity, seasonal monitoring, species-specific preventive measures, good record keeping, continuous surveillance, and education initiatives is essential.

## Authors’ Contributions

ABK: Collected data, performed statistical analysis, interpretation of results, and writing original draft. DNQ: Conceptualization, supervised the study, statistical analysis, interpretation of results, and extensively reviewed the manuscript. TS: Co-supervised the study and extensively reviewed and edited the manuscript. All authors have read, reviewed, and approved the final manuscript.
